# Instance-level localization and similarity-aware contrastive learning for robust plant disease classification

**DOI:** 10.3389/frai.2026.1913825

**Published:** 2026-10-29

**Authors:** Hira Batool, Mohsin Murad, Sultan Daud Khan, Amr Munshi, Majid Alotaibi, Muhammad I. Masud

**Affiliations:** 1 National University of Computer and Emerging Sciences FAST, Islamabad, Pakistan; 2 EjadTech, Riyadh, Saudi Arabia; 3 Faculty of Computing and Information Technology, Sohar University, Sohar, Oman; 4 Department of Computer and Network Engineering, College of Computing, Umm Al-Qura University, Makkah, Saudi Arabia; 5 Department of Electrical Engineering, College of Engineering, University of Business and Technology, Jeddah, Saudi Arabia

**Keywords:** deep learning, hybrid CNN, plant disease classication, weighted contrastive loss, YOLOv8n, lightweight

## Abstract

**Introduction:**

Plant disease classification in real-field images remains challenging due to complex backgrounds, overlapping leaves, multiple symptomatic regions, and visually similar disease categories. Conventional image based classifiers may therefore learn mixed representations in which disease-specific cues are confused with healthy tissue, background information, or co-occurring symptoms.

**Method:**

To address these challenges, this paper proposes an instance-aware and similarity-aware framework for plant disease classification. The proposed method first localizes disease using a lightweight YOLOv8n detector trained with coarse localization labels. Then patches corresponding to the localized regions are extracted and provided as input to hybrid CNN–Transformer architecture, where ResNet-18 captures fine-grained local lesion features and the transformer branch models broader contextual dependencies. These complementary representations are integrated through cross-attention fusion and projected into a shared embedding space. To improve inter-class separability, dynamically updated class prototypes are used to identify visually confusable disease pairs, and a Similarity-Weighted Contrastive Loss is introduced to put separation pressure on confused class boundaries. A decorrelation regularization term is further incorporated to reduce redundancy in the learned embeddings.

**Result:**

Experiments on the re-annotated DiaMOS Plant leaf dataset, evaluated under a source-image-level partitioning protocol that keeps all patches from the same field image within a single data partition, show that the proposed framework achieves 97.49 ± 0.73% accuracy and 94.82 ± 0.81% macro F1-score on a locked hold-out test set, significantly outperforming reimplemented baseline methods evaluated under the same experimental protocol (*p* < 0.001).

**Discussion:**

Embedding-space visualization using t-SNE and the normalized confusion matrix further demonstrate improved class separation and reliable prediction behavior across the four disease categories.

## 1 Introduction

Crop disease is one of the major threat to food security in the world. Various crop diseases lead to 20–40% loss in yield every year and cause billions of dollars in economic losses (Savary et al., 2012), especially in developing countries where economies are mainly based on agricultural (United Nations, 2019). Early and accurate diagnosis of plant diseases is thus important to control the damage (Kundu et al., 2022) by maintaining a balanced pesticide usage. Traditionally, plant disease diagnosis has relied on visual inspection by farmers or agricultural experts. Although expert-based diagnosis can be useful, it is time-consuming, subjective, dependent on human expertise, and difficult to scale over large agricultural regions (Upadhyay et al., 2025; Dong et al., 2023). These limitations have motivated the development of automated image-based disease recognition systems, where deep learning has emerged as a promising solution for fast, scalable, and cost-effective plant disease diagnosis (Hridoy et al., 2022; Mohanty et al., 2016).

In plant disease classification task, deep learning models have achieved remarkable success on benchmark datasets. Deep learning models, including MobileNet (Sinha and El-Sharkawy, 2019), Inception (Szegedy et al., 2016), and ResNet (He et al., 2016) have achieve good performance on potato disease classification (Jha et al., 2024). DenseNet-SVM hybrid (Bhola and Kumar, 2025) models and multi-scale convolutional models (Elfatimi et al., 2024) reported high performance on corn, wheat, and tomato datasets. Despite good performance of these model on various datasets, they share one common limitation associated. Most of these models are trained and evaluated on controlled data sets in which only one leaf of a plant is captured from frontal view with uniform background in the indoor environment. This setting is substantially different from the real-world agriculture settings, where multiple leaves, occlusion, variable illumination, complex background, multiple disease in one image can occur.

The real world dataset introduces the following practical challenges.

1. **Multi-disease co-occurrence:** The first challenge is the presence of more than one disease. In real-world agriculture setting, a single leaf might be infected with more than one disease or infected leaf might have more than one disease symptom (Khan et al., 2022; Jafar et al., 2024; Saleem et al., 2024). The majority of the models are tailored for simple background images. Models reported in Gautam et al. (2024); Dubey and Choubey (2024); Jha et al. (2024); Elfatimi et al. (2024) are trained and evaluated on single disease images with a simple background. Due to this reason, these models face challenges when applied to multi-disease classification scenarios. These models focus primarily on the most prominent visual pattern resulting in incorrect predictions. Some models have tried to tackle cluttered background by suppressing background, and introduce attention mechanism, like TC-MRSN (Wang et al., 2024) and DSTANet (Gao et al., 2025) for disease classification. These models do not explicitly localize the individual instances of the disease.
2. **Inter-class visual similarity:** The second challenge is inter-class similarity, where distinct diseases have similar appearance (e.g., structural similarity of lesions, chlorosis patterns, annd surface textures). This problem has been widely studied in literature. For example, Feng et al. (2024) reported considerable confusion between Cassava Mosaic Disease and Brown Streak Disease due to similarities in lesion characteristics. Similarly, wheat disease classification models (Shafi et al., 2021) failed to correctly classify Yellow Rust and Septoria, due to the presence of elongated chlorotic spot in both diseases. In soybean leaf disease diagnosis, (Sharma et al., 2025) highlighted the challenges to distinguish between early stages of different diseases, since different diseases appear significantly similar during the early stages. Similarly, work proposed by Upadhyay and Gupta (2024) also reports challenges in identifying the apple scab and rust in multiple crops.

Several attempts have been made to address the above mentioned challenges. One of the popular solution is the attention based mechanism. Attention mechanism guides the network to focus on disease-relevant features rather than treating the whole leaf uniformly. For example, Lee et al. (2020) presents RNN-based approach for plant disease classification. Similarly, Bera et al. (2024) proposes an attention-based network for plant disease classification. The network integrates high level CNN feature with attention mechanism to focus on discriminating regions of the leaf. Saini et al. (2022) develops an IoT-based plant disease monitoring system. The system is based on multi-input multi-output neural network integrated with attention mechanism to predict fungal disease. Wang et al. (2021) proposed an attention-base network with backbone of MobileNet. The network employs depthwise separable neural nework with Bayesian optimization for rice disease classification. Furthermore, Few-shot and metric-learning methods have also been introduced to improve inter-class similarity. Argüeso et al. (2020) presented few-shot learning for plant disease classification using deep Siamese networks.

Although the above mentioned methods have improved plant disease classification, they still do not fully address the two major challenges discussed above due to following reasons:

1. Although most of attention-based methods focus on disease-specific features, yet they still consider the image as a single classification unit. Therefore, when an image contains multiple diseases with multiple symptoms, these models may mix the visual information obtained from various parts of the image leading to incorrect classification.
2. Few-shot and metric-learning methods enhance feature separability by pushing apart the distance between disease classes in the embedding space, however, they apply the same separation constraint to all pairs of classes. As a result. they may unnecessarily split classes that are already well separated and fail to provide enough attention to visually similar classes.

In order to overcome these shortcomings, this study introduces a unified framework for plant disease classification. The framework employs instance-level disease localization and similarity-aware representation learning to overcome the above challenges. Generally, a lightweight YOLOv8n detector is trained to locate the regions of disease. Then patches corresponding to these regions and extracted. During this stage, we treat all the disease as one class. This avoids the problem of feature mixing due to the presence of multiple different regions with disease in the same image, and enables the classifier to work on one instance of the disease at a time. Then hybrid CNN-Transformer architecture is designed to extract local lesion texture, color variations, edge-level details as well as global contextual information from the every patch of the image. A cross-attention mechanism is used to fuse these complementary representations. Lastly, dynamically evolving class prototypes and a Similarity-Weighted Contrastive loss metric is utilized to select the most confusable disease pairs for training. In order to reduce the redundancy in the embedding space, also a decorrelation regularization term is employed. Therefore, the proposed framework directly tackles both the real-field multi-disease complexity and inter-class visual similarity to attain a more compact, separable and interpretable feature space for reliable plant disease classification.

Comparing to previous framework, proposed framework offer the following advantages and contributions:

- In contrast to previous conventional plant disease classification models which assign a single class label to whole image, the proposed framework handles multiple diseases by isolating each disease before the classification.
- The framework improves the inter-class similarity problem by introducing a similarity-weighted contrastive loss which separates visually similar disease classes by first identifying the most confusable class pairs and focuses optimisation to separate the difficult boundaries.
- A hybrid CNN-Transformer architecture is used to capture both local and global characteristics of the disease. The CNN branch extracts fine-grained details, while the transformer branch extracts long range contextual dependencies. The features from both branches are integrated through a cross-attention fusion mechanism which creates discriminative representations for visually similar diseases.
- The proposed framework is evaluated on the DiaMOS Plant leaf dataset and compared with several reimplemented baseline methods under a common experimental protocol. The results show that the proposed framework achieves higher mean accuracy and macro F1-score than the evaluated comparison methods.

The rest of the paper is organized as follows: Section 2 presents related literature on the classification of plant diseases, including approaches that deal with the multi-disease co-occurrence and inter-class visual similarity. Section 3 outlines the suggested methodology, including the detection-guided patch extraction approach, hybrid CNN-Transformer feature learning framework, and similarity-conscious optimization framework. The data set is discussed in Section 4. Section 5 describes the details of the training strategy and implementation. Section 6 discusses the experimental results and analysis, comparing it with the baseline techniques, ablation, and embedding space assessment. Section 7 concludes the paper.

## 2 Related work

### 2.1 Deep learning-based plant disease classification

Deep learning has become the dominant approach for plant disease classification because it can automatically learn disease-relevant visual features from leaf images without relying on hand-crafted descriptors. Several studies focused on using CNN for plant disease detection across different crops. Gautam et al. (2024) proposed an ensemble stacked deep neural network for classification of mango leaf diseases. Dubey and Choubey (2024) used color and texture descriptors and feature selection technique with MBi-LSTM classifier for recognizing paddy diseases. Jha et al. (2024) applied the stacking ensemble of MobileNet, Inception and ResNet for potato leaf disease classification and Deeba et al. (2024) explored a capsule-network-based disease monitoring system for muskmelon. Ghafar et al. (2025) applied deep transfer learning for the diagnosis of cotton leaf pathologies, reporting high classification accuracy across multiple disease categories.

While these methods have shown good performance, most of these methods follow whole-image classification approach, in which a single class label is assigned to the entire image. This formulation works well where there is one main leaf with clearly visual symptom. However, these methods may not be achieve reliable results in real-world scenarios, where, multiple leaves, complex backgrounds, occlusion, or multiple disese symptoms present. Moreover, a few models with high accuracy are tested primarily on controlled data sets (PlantVillage), where the background is simple and the disease is typically located in the center of the image. However, their performance may not reflect the challenges of the real-world scenarios. The DiaMOS Plant dataset was used by Alshammari et al. (2024) and improved the pear disease classification by using CycleGAN augmentation.

To improve robustness under complex visual conditions, several studies have introduced attention mechanisms, transformer architectures, and hybrid CNN–Transformer models. These methods aim to enhance feature discrimination by emphasizing disease-relevant regions, capturing multi-scale lesion patterns, or modeling long-range dependencies within leaf images. Wang et al. (2024) proposed TC-MRSN, a texture-color dual-branch network with multiscale residual shrinkage for maize leaf disease recognition under complex backgrounds. Gao et al. (2025) introduced DSTANet, which combines CNN and transformer components through decomposed spatial feature modeling and multi-scale token aggregation to suppress background noise and handle lesion-size variation. Khan et al. (2025) developed a Dense-Inception architecture with CBAM attention to improve feature discrimination under inter-class similarity and intra-class variability. Similarly, Sharma et al. (2025) proposed SoyaTrans, a CNN–Swin Transformer framework for fine-grained soybean disease classification, while Xiang et al. (2025) introduced DWTFormer to combine frequency-domain and spatial features for tomato leaf disease identification.

Region-focused disease recognition methods have also been explored through object-detection-based approaches. Fuentes et al. (2017) used deep learning detectors for real-time tomato disease and pest recognition in images captured under practical conditions. Singh D. et al. (2020) introduced PlantDoc, a real-world plant disease detection dataset containing annotated images across multiple plant species and disease categories. In recent years, the direct use of YOLOv8 detectors in plant diseases recognition has been proposed. Ghafar et al. (2024) fine-tuned a custom YOLOv8 model for single-stage detection and classification stages, and tested its transferability on external images of plant conditions. The fine-grained disease classification is done within the detector itself, and in these approaches, localization and category discrimination are jointly optimized in one detector (multi-class detection head). For the detector, there is thus a need to distinguish between disease classes that are visually similar only by their box-level features, and the more diseases that are added, the more classes the detector must distinguish between. The proposed framework is different: all disease categories are combined into a single foreground class in the detection stage, and a new patch-level disease category classifier modeled by similarity-aware optimization is used to classify the disease category. This decoupling enables similarity estimation in the prototyping method and separation of similarity and contrastiveness in the localized instances, which is not possible when the classification is performed within the detection head. These studies reveal the need to go beyond image level classification and toward a more realistic disease localization and recognition setting.

However, most attention and transformer based classification models process the complete image before generating the final prediction. Although attention mechanism can suppress irrelevant regions, and transformers can capture broader spatial dependencies, these mechanisms do not necessarily separate individual disease instances before feature learning. Consequently, if multiple symptomatic regions or leaves are present in the same image, the extracted representation may still combine disease evidence, healthy tissue, and background information into single global descriptor. In contrast, the proposed framework first constructs localized disease or healthy leaf patches before classification, thereby reducing input-level feature mixing before applying similarity-aware representation learning.

Several studies have attempted to address inter-class similarity problem through metric learning (Lakehal et al., 2024), (Singh V. et al., 2020), prototype similarity (Liang et al., 2024), contrastive learning (Mu et al., 2024), or class-separation losses (Uzhinskiy, 2025). Egusquiza et al. (2022) analyzed few-shot and metric-learning strategies, including contrastive and triplet-based losses, for fungal plant disease classification and showed that embedding-space learning can improve class separability. Sunday et al. (2025) introduced a Weighted Cluster Loss to reduce overfitting and improve inter-class separation in tomato disease severity classification. Yang et al. (2022) explicitly evaluated inter-class similarity using prototype distances and used this information to identify informative samples for plant disease classification. Xiao et al. (2025) proposed class-agnostic contrastive learning with supervised organizational mapping to improve inter-class feature separation in citrus leaf disease recognition. Kaur et al. (2024) also addressed disease separability through a hybrid CNN-based framework that combines convolutional feature extraction, attention, and SVM-based classification.

Prototypical networks were introduced by Snell et al. (2017) to learn the prototypes of each class. Supervised contrastive learning (Khosla et al., 2020) was introduced as a method to learn samples belonging to the same class and separate samples from different classes. Zbontar et al. (2021) introduced the concept of redundancy reduction to mitigate embedding dimensions that lead to redundant representations and to enhance the quality of the representation. These methods inspire the proposed framework to adopt prototype-based similarity estimation, similarity-weighted contrastive learning and decorrelation regularization.

## 3 Methodology

### 3.1 Overview of the proposed framework

The proposed framework handles plant disease classification on two complementary levels. At the input level, it converts images into localized instance-level patches so that the classifier receives cleaner visual evidence. At the embedding level, it improves the separability between visually similar classes by applying higher penalty on the classes. The entire workflow is shown (Figure 1).

**Figure 1:**
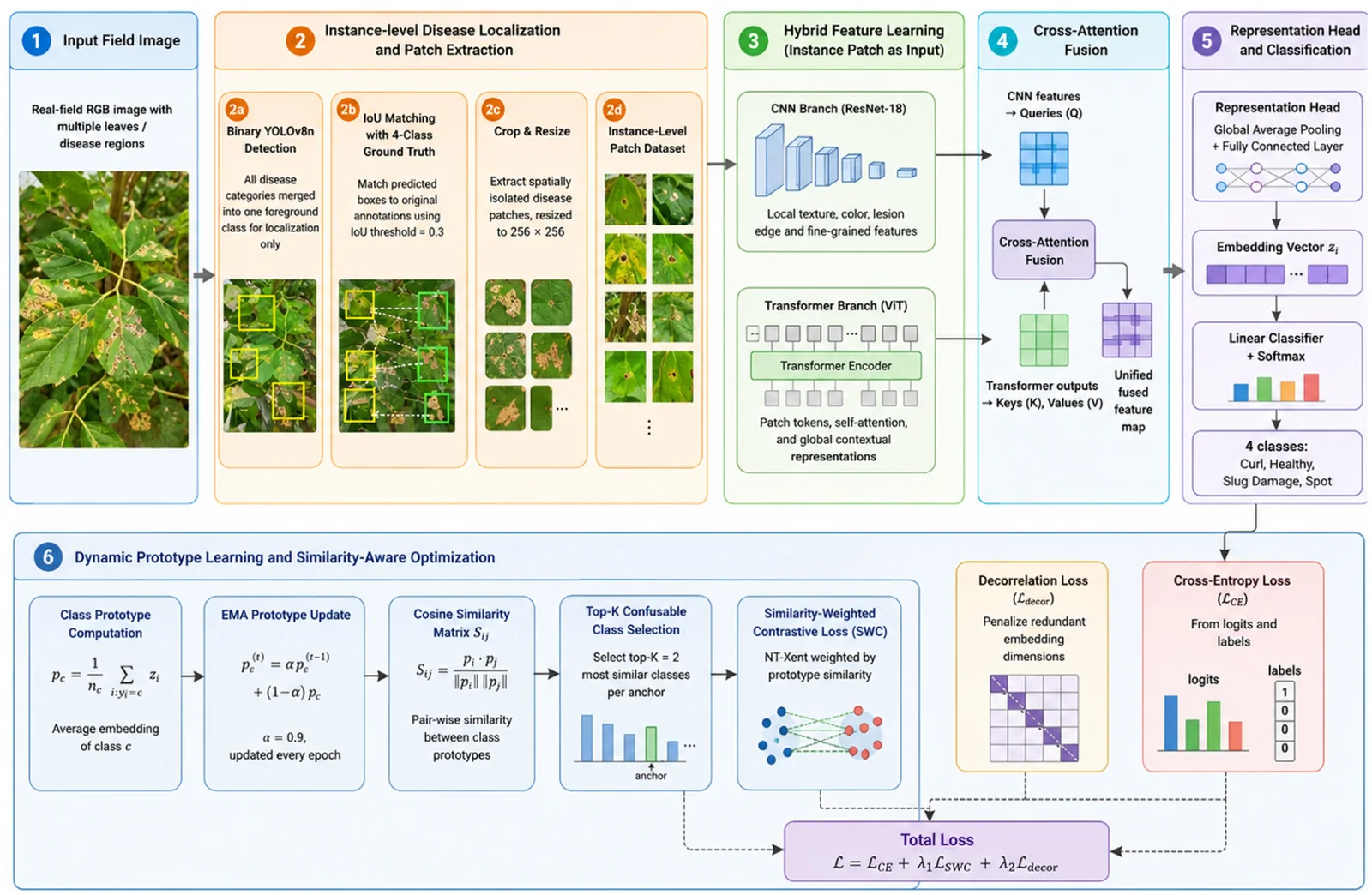
Overview of the proposed instance-aware and similarity-aware plant disease classification framework.

The framework consists of three main stages. First a lightweight YOLO model is used to identify and extract the lesion region. These regions are then converted to sample patches which are processed by a hybrid CNN-Transformer feature extractor, where convolutional features capture local lesion patterns and transformer features capture broader contextual dependencies. These representations are then fused using cross-attention to obtain a shared embedding. Third, the learned embeddings are improved using a similarity-based contrastive loss that is guided by class prototypes that are updated during training. Cross-entropy loss is also used to directly guide the model toward the correct class, while decorrelation regularization helps reduce repeated or unnecessary information in the embeddings. The final embedding is then used for class prediction and for learning representations based on similarity.

### 3.2 Problem formulation

Let $D=\left(I_{i},Ai\right){i=1}^{N}$ represent the original image-level dataset, where *I*_*i*_ is a real-field RGB image and $Ai=\left(bij,y_{ij}\right){j=1}^{M_{i}}$ contains the annotated regions in image *I*_*i*_. Here, *bij* is the bounding box of the *j*-th annotated region, while *y*_*ij*_∈1, 2, …, *C* is its class label. The value *C* represents the total number of target classes, and *M*_*i*_ is the number of annotated regions in image *I*_*i*_. In conventional whole-image classification, each image is treated as a single training sample. However an image taken in real-field scenario may contain multiple leaves with various diseases, healthy tissue, background clutter, and several symptomatic regions. So training directly such images may introduce a mixture of different visual information into the learned features.

To address this issue, the proposed framework treats plant disease classification as an instance-level learning problem. Instead of assigning a single label to the entire image, the original dataset is converted into a patch-level dataset using Equation 1.


$$\begin{array}{l} D_{p}={\left(x_{n},y_{n}\right)}_{n=1}^{N_{p}}, \end{array}$$ (1)

where *x*_*n*_ denotes a localized disease or healthy leaf patch and *y*_*n*_ is its corresponding class label. Here, *N*_*p*_ represents the total number of localized patches generated from the original image-level dataset. The goal is to train a classifier that predicts the class of each localized patch instead of the class of complete field image. This will help model to focus on the most relevant visual information in each region and reduces the effect of irrelevant background content.

### 3.3 Disease instance localization and patch extraction

The first stage constructs instance-level samples from the original field images. A lightweight YOLOv8n-based localization model is used to identify relevant plant regions before classification. The detector is trained using coarse localization labels rather than fine-grained disease labels. In this stage, visually diseased regions are localized as disease-region instances, while representative healthy leaf regions are also used to support the healthy class in the patch-level classification setting. This separates the localization task from the fine-grained disease classification task: the detector identifies where relevant plant regions are located, and the classifier later determines the final class of each cropped patch. The locked test images are separated at the source-image level before patch extraction, ensuring that patches originating from the same source image remain within the same data partition. The detector is trained using only the development pool images, so the locked test images are unseen by both the localization and the classification stages. For patch construction in the development pool, each predicted bounding box is matched with the original four-class annotation using intersection-over-union (IoU). The predicted region is assigned the class label of the ground-truth annotation with the highest IoU if the matching score satisfies the threshold of 0.3. A threshold of 0.3 is used rather than the conventional 0.5 because lesion regions are small and irregular, so a stricter criterion would discard a large number of correctly localized symptomatic regions. For the development pool, regions that do not satisfy this criterion are discarded because a ground-truth class label is required for classifier training.

The matched regions are then cropped from the original image and resized to 256 × 256, from which 224 × 224 classifier inputs are obtained by random cropping during training and center cropping at evaluation. In this way, not the entire field image is passed to the classifier, but just the areas of disease or healthy leaves, which minimizes any feature mixing that can occur from background leaf areas, overlapping leaves, or multiple diseased areas.

For the locked test images, the detector predictions are processed without using ground-truth bounding boxes. Detected regions are cropped and passed directly to the classifier, including detections that do not match a ground-truth region. Thus, the test-time pipeline performs localization followed by classification without access to ground-truth bounding boxes. Images for which the detector produces no region are retained as detection failures in the end-to-end evaluation.

Since unmatched detections carry no ground-truth class label, the patch-level classification metrics reported in Section 6 are computed over the 1,315 labeled test patches. The remaining 426 detected regions are retained within the locked test partition for completeness but are excluded from the labeled-patch metrics.

### 3.4 Hybrid feature learning with cross-attention fusion

After localizing the extracted patch, each patch is fed to a hybrid CNN-Transformer feature learning module. CNN branch is based on ResNet-18 and is used for the detection of local features of the disease such as lesion boundaries, spot texture, color variations, and fine-grained edge patterns. These local details are important because diseases that look alike can have only slight variations in the features of the symptoms.The ResNet-18 backbone is pretrained on ImageNet and the last classification layers are discarded. A 1 × 1 convolution is then applied to project the resulting CNN feature map to 512 channels. The transformer branch simultaneously works on the same patch as a series of image tokens and learns long-range spatial relationships using self-attention. This enables the model to capture more general context relationships in the local area of the plant. The transformer branch operates on 224 × 224 input patches using 16 × 16 tokenization patches. This produces 196 image tokens and ach token is represented using a 512-dimensional embedding. Transformer consists of four encoder layers, with eight attention heads and a feed-forward dimension of 1024.

Let $F_{n}^{cnn}$ and $F_{n}^{tr}$ denote the feature representations extracted from the CNN and transformer branches for patch *x*_*n*_, respectively. In order to combine these representations a cross-attention fusion module is used. The CNN features are projected as queries and the transformer features are used as keys and values. The attended representation and final embedding are computed as in Equation 2


$$\begin{array}{lll} A_{n} & = & \mathrm{softmax}\left(\frac{\left(F_{n}^{cnn}W_{Q}\right){\left(F_{n}^{tr}W_{K}\right)}^{T}}{\sqrt{d_{h}}}\right)\left(F_{n}^{tr}W_{V}\right),\\ z_{n} & = & g\left(\mathrm{Fuse}\left(F_{n}^{cnn},A_{n}\right)\right), \end{array}$$ (2)

where *W*_*Q*_, *W*_*K*_, and *W*_*V*_ are learnable projection matrices, *d*_*h*_ is the feature dimension of each attention head, and *A*_*n*_ is the cross-attended representation. There are 8 heads in cross attention module. The spatial positions of the CNN feature map are first flattened and used to create the query, the transformer tokens serve as keys and values. The output of all heads are concatenated and fed into a learnable output projection *W*_*O*_. The fusion operation is a residual fusion between the projected attention output and the CNN feature map, as in Equation 3


$$\begin{array}{l} \mathrm{Fuse}\left(F_{n}^{cnn},A_{n}\right)=\mathrm{LayerNorm}\left(F_{n}^{cnn}+W_{O}A_{n}\right), \end{array}$$ (3)

so that the globally contextualized information carried by *A*_*n*_ is added to the local convolutional representation. This helps keep the fine-grained lesion detail while also giving long-range context. The *g*(·) denotes the representation head consisting of global pooling and a fully connected projection layer. Global average pooling is then applied to the fused feature map, followed by a fully connected layer that produces a 256-dimensional embedding. The resulting embedding *z*_*n*_ is used for both final classification and similarity-aware contrastive learning. A linear classifier is applied on top of *z*_*n*_ to generate the class logits used by the cross-entropy loss.

### 3.5 Similarity-weighted contrastive learning

The last stage enhances the embedding space organization by learning the visually confusing disease classes. In general, conventional contrastive learning assumes that the negative class pairs have equal visual similarity, which is not the case for the plant disease classes. Some class pairs are already well separated and others have similar lesion shape, color, texture or symptom structure. To compensate for this, the proposed framework dynamically estimates the similarity between classes by class prototypes and applies the strongest contrastive pressure to the most similar class pairs.

For each class *c*, a prototype is computed from the embeddings of samples belonging to that class. At the end of every training epoch, the current prototype estimate ${\hat{p}}_{c}$ is obtained as the mean embedding of all training samples of class *c* in the corresponding fold, computed in evaluation mode. Since the embedding space changes throughout training, the stored prototype is then updated using an exponential moving average, which stabilizes the estimate against epoch-to-epoch fluctuation. Pairwise class similarity is then estimated using cosine similarity using Equation 4:


$$\begin{array}{lll} {\hat{p}}_{c} & = & \frac{1}{N_{c}}\underset{n:y_{n}=c}{\sum }z_{n},\;p_{c}^{\left(t\right)}=\alpha p_{c}^{\left(t-1\right)}+\left(1-\alpha \right){\hat{p}}_{c}^{\left(t\right)},\\ S_{c,d} & = & \frac{p_{c}^{T}p_{d}}{||p_{c}|{|}_{2}||p_{d}|{|}_{2}}. \end{array}$$ (4)

Here, ${\hat{p}}_{c}$ is the current prototype estimate, $p_{c}^{\left(t\right)}$ is the prototype of class *c* at epoch *t*, α is the momentum coefficient, and *S*_*c, d*_ denotes the similarity between classes *c* and *d*. In this study, α is set to 0.9. The prototypes are initialized directly from the class-wise mean embeddings obtained after the first training epoch, and the moving average is applied from the second epoch onwards. The contrastive term is therefore inactive during the first epoch, when the embedding space is not yet informative. For each class, the top-*K* most similar classes are selected from the similarity matrix and treated as the confusable class set $K_{c}$. In this study, *K* = 2.

For each training mini-batch, the confusable class pairs are determined from the classes included in that mini-batch. Up to 8 samples are randomly chosen from each class to form the pair-wise contrastive objective for each selected pair. If the selected confusable class is not in the current mini-batch, the pair is skipped and the contrastive loss is calculated from the remaining valid pairs. If there is no valid confusable pair, then the value of the contrastive contribution is set to zero.

For a selected pair of classes (*c, d*), let *M* embeddings be sampled from each class and normalized to unit length, giving a contrastive set of 2*M* embeddings. For an anchor *z*_*i*_ in this set, the positive set P(i) contains the remaining embeddings of the same class as *z*_*i*_, while the embeddings of the opposing class act as negatives. The pair-wise objective is the supervised normalized temperature-scaled cross-entropy loss computed as in Equation 5.


$$\begin{array}{l} L_{\text{pair}}\left(c,d\right)=-\frac{1}{2M}\overset{2M}{\underset{i=1}{\sum }}\frac{1}{\left|P\left(i\right)\right|}\underset{q\in P\left(i\right)}{\sum }\log\frac{\exp\left(z_{i}^{T}z_{q}/\tau \right)}{\sum_{k\neq i}\exp\left(z_{i}^{T}z_{k}/\tau \right)}, \end{array}$$ (5)

where τ is the contrastive temperature. Minimizing this objective increases the similarity of same-class embeddings while decreasing the similarity between the two selected classes, so the pair (*c, d*) is driven apart in the embedding space.Since the objective is to concentrate separation effort on the class pairs that are hardest to distinguish, the pair-wise weight increases monotonically with prototype similarity. The cosine similarity is first mapped from [−1, 1] to [0, 1] so that the weight remains non-negative, giving by Equation 6


$$\begin{array}{l} \omega_{c,d}={\left(\frac{1+S_{c,d}}{2}\right)}^{\gamma }, \end{array}$$ (6)

where γ controls the effect of the similarity-based weighting and is set to 1 in this study. Class pairs whose prototypes lie close together in the embedding space therefore receive a proportionally larger contrastive weight, while pairs that are already well separated contribute less to the objective. Together with the top-*K* selection, this ensures that optimization effort is concentrated on the most confusable decision boundaries rather than distributed uniformly over all class pairs. The similarity-weighted contrastive loss is obtained as by using Equation 7


$$\begin{array}{l} L_{SWC}=\frac{1}{\left|V\right|}\underset{\left(c,d\right)\in V}{\sum }\omega_{c,d}L_{\text{pair}}\left(c,d\right), \end{array}$$ (7)

where V denotes the set of valid confusable class pairs available in the current mini-batch.

The contrastive temperature is set to τ = 0.1, and the maximum number of samples used from each selected class pair is set to eight. To reduce redundancy among embedding dimensions, a decorrelation regularization term is applied to the batch embedding matrix $Z\in {\mathbb{R}}^{B\times d_{z}}$ after mean normalization. The covariance matrix, decorrelation loss, and final training objective are written compactly as Equation 8 and Equation 9


$$\begin{array}{l} C_{Z}=\frac{1}{B-1}Z^{T}Z,\;L_{decor}=\frac{1}{d_{z}}\underset{r\neq s}{\sum }{\left(C_{Z}\right)}_{r,s}^{2}, \end{array}$$ (8)

$$\begin{array}{l} L_{total}=L_{CE}+\lambda_{1}L_{SWC}+\lambda_{2}L_{decor}. \end{array}$$ (9)

The cross-entropy loss $L_{CE}$ maintains direct classification supervision, the similarity-weighted contrastive loss $L_{SWC}$ strengthens the separation of visually confusable classes, and the decorrelation term $L_{decor}$ encourages different embedding dimensions to encode complementary information. The cross-entropy loss uses inverse-frequency class weights computed from the training fold only, so that the class imbalance of the patch dataset does not bias the supervised term. Together, these objectives guide the model toward an embedding space that is compact within classes, separated between confusable classes, and less redundant across feature dimensions.

## 4 Dataset

This research uses DiaMOS Plant dataset (Fenu and Malloci, 2021) which is composed of the RGB images obtained by a smartphone camera and DSLR camera. This work considers only leaf-based disease classification, with the original dataset comprising of leaf and fruit images. Thus, only the leaf images were chosen for the experiments. There are four categories in the selected subset: curl, healthy, slug damage, and spot. A total of 3006 leaf images were used including 43 healthy, 884 spot, 54 curl and 2025 slug damage images. There is a high class imbalance in this distribution, with most samples being slug damaged or spot, and a relatively small number of healthy and curl. Figure 2 presents the original dataset structure used in this study, including representative samples from the four classes and the annotation format provided by the DiaMOS Plant dataset.

**Figure 2:**
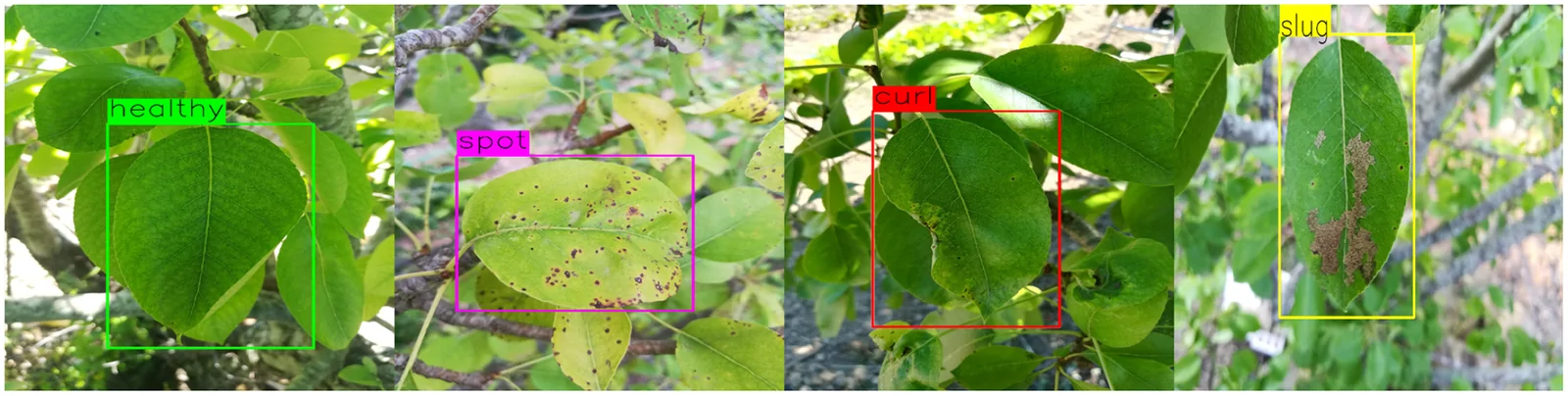
Original DiaMOS Plant leaf dataset representation used in this study, showing the four selected classes and the annotation format provided by the dataset.

The DiaMOS Plant dataset is well suited for evaluating plant disease classification under real-field conditions because its images were captured in uncontrolled outdoor environments during the growing season. The dataset contains natural variations in illumination, camera viewpoint, leaf orientation, background clutter, symptom size, occlusion, and disease severity.

Although the original DiaMOS annotations provide useful image-level class information, they are limited for the objective of this study because each image is assigned a dominant label rather than separate labels for individual symptomatic regions. To support the proposed instance-level framework, the selected leaf images were re-annotated using bounding boxes around visible disease regions. For the healthy class, representative healthy leaf regions were also annotated to maintain a consistent patch-level formulation across all four classes. This re-annotation converts the original image-level dataset into an instance-level dataset suitable for detection-guided classification, as shown in Figure 3.

**Figure 3:**
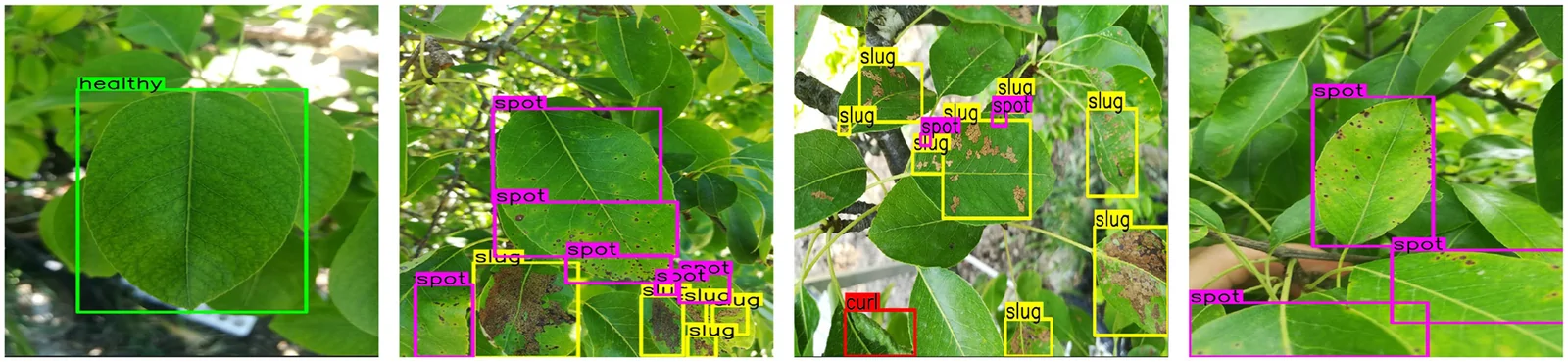
Re-annotated dataset samples with bounding boxes marking individual disease instances within a single image.

Following localization, the selected images were converted into an instance-level patch dataset containing a total of 9,761 localized patches, including 8,020 patches from the development pool and 1,741 patches from the locked test set. The development pool patches included 189 curl, 189 healthy, 4,732 slug damage, and 2,910 spot patches. The locked test set contained 25 curl, 31 healthy, 810 slug damage, and 449 spot patches with ground-truth matches, while an additional 426 detected regions did not satisfy the IoU matching criterion and were retained for test-time inference. The development pool and locked test set contained 2,555 and 451 source images, respectively, with an average of 3.14 and 3.86 localized patches per image.

## 5 Experimental setup

All experiments were conducted using PyTorch and run on Kaggle cloud on NVIDIA default GPUs. To ensure reproducibility, a fixed random seed of 42 was used. The training pipeline was as follows: (1) YOLOv8n was trained on the re-annotated bounding-box dataset with coarse localization labels, and (2) instance-level patches were cropped and fed to the proposed hybrid CNN–Transformer classifier.

The test set and development pool were first split at image level using stratified sampling with a fixed random seed of 42, creating 15% test set and 85% development pool. The detector was trained only using the development images, so the test images were kept completely separate. The development images were divided into 5 folds, while keeping all patches from the same image in the same fold. The classifier was trained a maximum of 30 epochs with early stopping on. The validation macro-F1 is used and the patience of five epochs is adopted. Only the training partition was augmented. The locked test set was not involved in training or model selection, and only used for the final evaluation. Following results are reported on the test set that is locked. Each of the five models was evaluated separately providing a mean and standard deviation that indicate fold-to-fold variation and an ensemble prediction was used based on averaging the logits across the five models. Values are presented the mean ± sample standard deviation, fold-wise.

All reimplemented comparison methods were trained and evaluated under exactly this protocol. Each method used the same source-image split, the same five folds, the same locked test set, and the same optimizer, learning-rate schedule, epoch budget, early-stopping criterion, input resolution, augmentation, normalization statistics, and class-imbalance handling as listed in Table 1; only the network architecture and the training objective differed. No per-method hyperparameter search was carried out, so the comparison reflects how each design performs under a common training budget rather than the best achievable result for each method. The main implementation and training configurations are summarized in Table 1.

**Table 1:** Summary of the experimental configuration used in the proposed framework.

Component	Configuration
Implementation framework	PyTorch
Execution platform	Kaggle cloud GPU
Random seed	42
Detector	YOLOv8n with pretrained weights
Detector input size	640 × 640
Detector epochs / batch size	50 / 16
Detector training data	85% development pool only
Detector validation split	80% / 20% of the development pool
Localization labels	Coarse disease-region / healthy-region labels
Detection confidence threshold	0.3
IoU matching threshold	0.3
Locked test set	15% of source images, stratified
Classifier development protocol	Stratified grouped 5-fold CV
Grouping variable	Source image
Extracted patch size	256 × 256
Classifier input size	224 × 224
Optimizer	AdamW
Learning rate / weight decay	1 × 10^−4^ / 1 × 10^−4^
LR scheduler	Cosine annealing
Classifier epochs / batch size	30 / 32
Gradient clipping	5.0
Class imbalance handling	Inverse-frequency class weights from the training fold
Training augmentation	Random crop, horizontal and vertical flips, color jitter
Normalization	ImageNet mean and standard deviation
Early stopping	Validation macro-F1, patience 5
Prototype momentum α	0.9
Top-*K* similar classes	2
Contrastive temperature τ	0.1
Samples per class pair	8
Similarity weight exponent γ	1.0
Loss weights	λ_1_ = 0.5, λ_2_ = 0.1
Ensemble strategy	Logit averaging

## 6 Results and discussion

### 6.1 Evaluation protocol

The accuracy and macro F1-score were used as the performance measures for the proposed framework. All the comparison methods were reimplemented and trained under the same protocol as described in Section 5 with the same source-image split, the same five-folds and the same locked test set, with the following results. All results reported in this section are on the locked hold-out test set that was not used for training or model selection. The five fold models were tested separately on the locked test set for each method and the results are given as mean ± sample standard deviation. This standard deviation is not a reflection of the uncertainty of the test data since all five models are tested on the same images. The comparison thus reflects the average performance of each method as well as the consistency of performance across training partitions.

### 6.2 Comparison with re-implemented inter-class similarity methods

Table 2 compares the proposed framework with the re-implemented methods. The purpose is not to reproduce the results these methods reported on their own datasets, but to see how each design performs on the DiaMOS Plant leaf dataset when every method is given the same data, the same folds, and the same training budget.

**Table 2:** Quantitative comparison of the proposed framework with re-implemented baseline methods in terms of accuracy and macro F1-score.

Ref.	Author/year	Accuracy	Macro F1-score
Gao et al. (2025)	Gao et al. (2025)	83.33 ± 3.77	59.64 ± 16.94
Xiang et al. (2025)	Xiang et al. (2025)	73.69 ± 3.91	59.61 ± 6.67
Kaur et al. (2024)	Kaur et al. (2024)	89.36 ± 1.51	82.75 ± 5.04
Sunday et al. (2025)	Sunday et al. (2025)	86.16 ± 5.41	43.91 ± 0.47
Khan et al. (2025)	Khan et al. (2025)	58.09 ± 10.24	34.32 ± 11.46
Egusquiza et al. (2022)	Egusquiza et al. (2022)	89.93 ± 1.34	87.08 ± 1.60
Xiang et al. (2025)	Xiang et al. (2025)	79.47 ± 3.60	44.71 ± 7.80
–	**Proposed framework**	**97.49** **±** **0.73**	**94.82** **±** **0.81**

Values are reported as mean ± standard deviation across the five fold models, all evaluated on the locked hold-out test set. The values in bold indicate the best results among the compared methods.

Egusquiza et al. (2022) is the strongest baseline, with 89.93 ± 1.34% accuracy and 87.08 ± 1.60% macro F1-score. The proposed framework achieves 97.49 ± 0.73% accuracy and 94.82 ± 0.81% macro F1-score giving an improvements of 7.56 and 7.74 percentage points. The Welch *t*-test confirms that this difference is larger than the variation between folds for both accuracy (*t* = 11.08, *p* < 0.001) and macro F1-score (*t* = 9.65, *p* < 0.001). Most of the comparison methods show much higher accuracy than macro F1-score. For example Sunday et al. (2025) achieves 86.16 ± 5.41% accuracy but only 43.91 ± 0.47% macro F1-score, and both Xiang et al. (2025) variants show a similar pattern. A model can have high accuracy by correctly predicting majority classes like slugh and spot while performing poorly on minority classes such as curl and healthy in this case. Macro F1-score avoids this imbalance by giving equal importance to all four classes, making it a useful metric for imbalanced dataset like this one.

There are also significant variations in stability between folds. The proposed framework is much more stable with a difference of not more than one point for macro F1-score whereas Gao et al. (2025) and Khan et al. (2025) deviate by more than 10% points in macro F1-score, depending on the fold. The whole image is processed by the baseline methods, which include background, healthy areas, and different symptoms before attempting to separate the classes. As a consequence, their outcomes may vary significantly based on the images utilized for training. The variation at the input stage is reduced in the proposed framework, as each patch has one disease instance. This provides the contrastive learning step with cleaner information and results in more stable results. This is the central theme of the study and the improvements over methods that primarily address class similarity via features or loss functions validates this claim.

### 6.3 Ablation study

To analyze the contribution of different components, an ablation study was conducted using different architectural and loss configurations. We conduct the ablation study using the following four configurations in addition to the proposed method: 1) CNN only (ResNet-18) + CE loss, 2) Transformer only (ViT) + CE loss, 3) ResNet-18 + ViT + CE loss, 4) ResNet-18 + ViT + CE loss + SWC loss. The first four configurations receive the complete field image as input, while the proposed framework receives localized instance-level patches, so the comparison isolates the effect of the input representation as well as the architectural and loss components. The results are shown in Table 3. The CNN-only and transformer-only configurations achieve comparable performance, with the ResNet-18 model obtaining 0.882 accuracy and 0.869 macro F1-score, while the ViT model obtains 0.867 accuracy and 0.866 macro F1-score. The results show that local convolutional features, along with transformer-based contextual features, are both helpful for this dataset, but neither type is enough to completely overcome the classification challenge. Interestingly, it is observed that the accuracy of the combined model ResNet-18 and ViT with the cross entropy loss is not improved, but it is reduced to 0.860 and the macro F1 score is slightly increased to 0.874. This indicates that the use of local and global features is not enough to improve the classification, if the input representation and the optimization goal are not appropriately designed.

**Table 3:** Ablation study showing the effect of different architectural and optimization configurations on accuracy and macro F1-score.

Experiment	Input	Accuracy	Macro F1-score
CNN only (ResNet-18) + CE loss	Whole image	0.882	0.869
Transformer only (ViT) + CE loss	Whole image	0.867	0.866
ResNet-18 + ViT + CE loss	Whole image	0.860	0.874
ResNet-18 + ViT + CE loss + SWC loss	Whole image	0.823	0.856
**Proposed framework**	**Patch**	**0.980**	**0.939**

All values are obtained on the locked hold-out test set using the logit-averaged ensemble of the five fold models. The first four configurations use whole-image input; the proposed framework uses localized instance-level patches. The values in bold indicate the best results among the compared methods.

The addition of the Similarity-Weighted Contrastive loss to the whole-image hybrid representation further reduces performance to 0.823 accuracy and 0.856 macro F1-score. This result is important because it shows that a stronger metric-learning objective is not automatically beneficial when the underlying input representation still contains mixed visual information. If the model receives full images containing multiple leaves, background clutter, and mixed symptomatic regions, contrastive separation may become unstable because the embeddings do not correspond to clean disease instances. Therefore, the ablation study indicates that embedding-level separation alone cannot fully compensate for input-level ambiguity.

The complete proposed framework achieves a substantial improvement, reaching 0.980 accuracy and 0.939 macro F1-score. The large gain demonstrates that the most important design choice is the reformulation of the classification problem from whole-image learning to instance-level disease-region learning. Once the classifier receives localized patches, the hybrid CNN-Transformer representation and similarity-aware optimization can operate on cleaner disease-specific features. Thus, the ablation results show that the strength of the proposed method does not come from adding architectural components alone, but from combining instance-level localization with similarity-aware representation learning.

The values reported here are ensemble predictions on the locked test set and therefore differ slightly from the fold-wise means in Table 2, where each fold model is scored individually.

Figure 4 shows the comparisons of different configurations.

**Figure 4:**
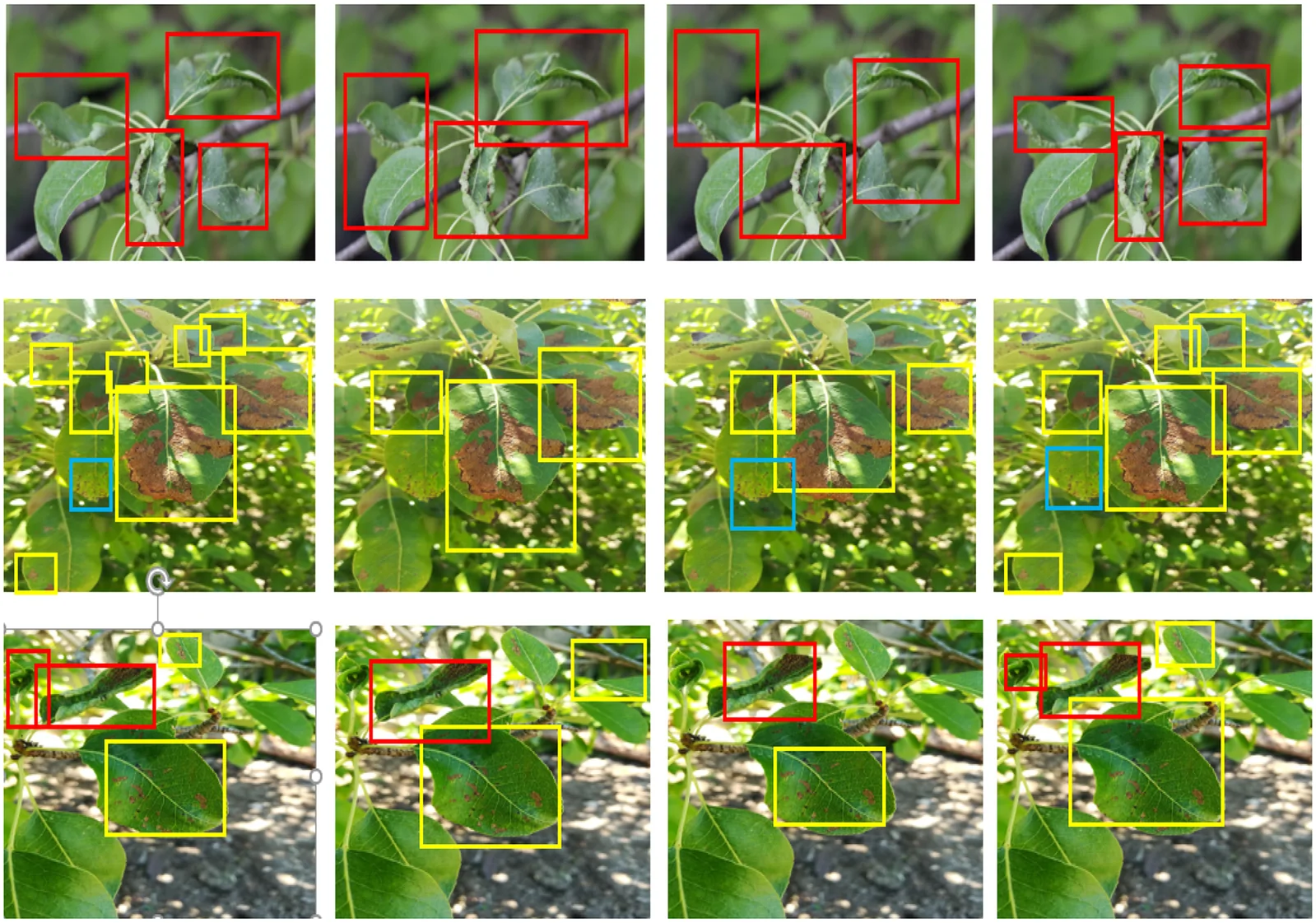
Visualization of prediction results of different configurations. The first column represents the sample image with ground truth bounding boxes. The second column represents the results of ResNet-18 + ViT + CE loss, third column represents the results of CNN only (ResNet-18) + CE loss, and fourth column shows the results of proposed framework. Red color represents “curl,” yellow color represents “slug,” and cyan represents “spot.”

### 6.4 Per-class performance analysis

Table 4 reports the class-wise F1-scores for each experimental configuration. This analysis is significant because the dataset is highly unbalanced, and accuracy could be misleading if the performance on minority or difficult-to-identify classes is under-performing.

**Table 4:** Per-class F1-scores for all experimental configurations.

Experiment	Curl (*n* = 25)	Healthy (*n* = 31)	Slug (*n* = 810)	Spot (*n* = 449)
CNN only (ResNet-18) + CE loss	0.88	0.85	0.91	0.81
Transformer only (ViT) + CE loss	0.84	0.92	0.90	0.79
ResNet-18 + ViT + CE loss	0.88	0.92	0.89	0.79
ResNet-18 + ViT + CE loss + SWC loss	0.88	0.85	0.89	0.78
**Proposed framework**	**0.82**	**0.97**	**0.99**	**0.98**

The number of locked test patches available for each class is given in the header. The values in bold indicate the best results among the compared methods.

The baseline configurations show uneven class-wise behavior. In particular, the spot class obtains the lowest F1-scores across several configurations, ranging from 0.78 to 0.81. This indicates that the class remains difficult to classify when the model is trained using conventional full-image or non-localized representations. The healthy and curl classes also show variation across experiments, reflecting the difficulty caused by limited samples and visual overlap with other categories.

The proposed framework raises the F1-score for healthy, slug, and spot to between 0.97 and 0.99. The gain on spot is the clearest, moving from 0.78–0.81 in the baseline configurations to 0.98, which matters because this class was consistently the weakest before localization was introduced. Slug and spot together account for the majority of the test patches, so these two classes largely determine the overall accuracy reported in Table 3.

The curl class behaves differently. Its F1-score of 0.82 is the lowest of the four and falls below the 0.88 obtained by the CNN-only and hybrid baselines. The precision and recall for this class are 0.83 and 0.81, so the errors are split between missed curl patches and other classes predicted as curl, rather than concentrated on one side. Curl is represented by only 25 patches in the locked test set, which places a 95% confidence interval of roughly [0.61, 0.91] on its recall. The corresponding interval for slug, with 810 test patches, spans about [0.98, 0.99]. The curl figure is therefore an unstable estimate, and the difference between 0.82 and the 0.88 of the baseline configurations is well inside the range that this sample size can resolve. Curl is also the rarest class in the original dataset, with 54 source images, so few examples are available at either training or test time.

Overall, the proposed framework gives balanced performance on three of the four classes and does not rely on the majority classes alone to raise its aggregate scores. Curl remains the weakest category, and reliable evaluation of this class would require a larger annotated sample.

### 6.5 Confusion matrix and embedding-space analysis

To complement the overall accuracy and macro F1 score assessment of the proposed framework, two additional analyses were conducted: embedding space visualization and prediction-level confusion analysis. The learned test embeddings are plotted in Figure 5a, and the normalized confusion matrix is displayed in Figure 5b, based on the final predictions.

**Figure 5:**
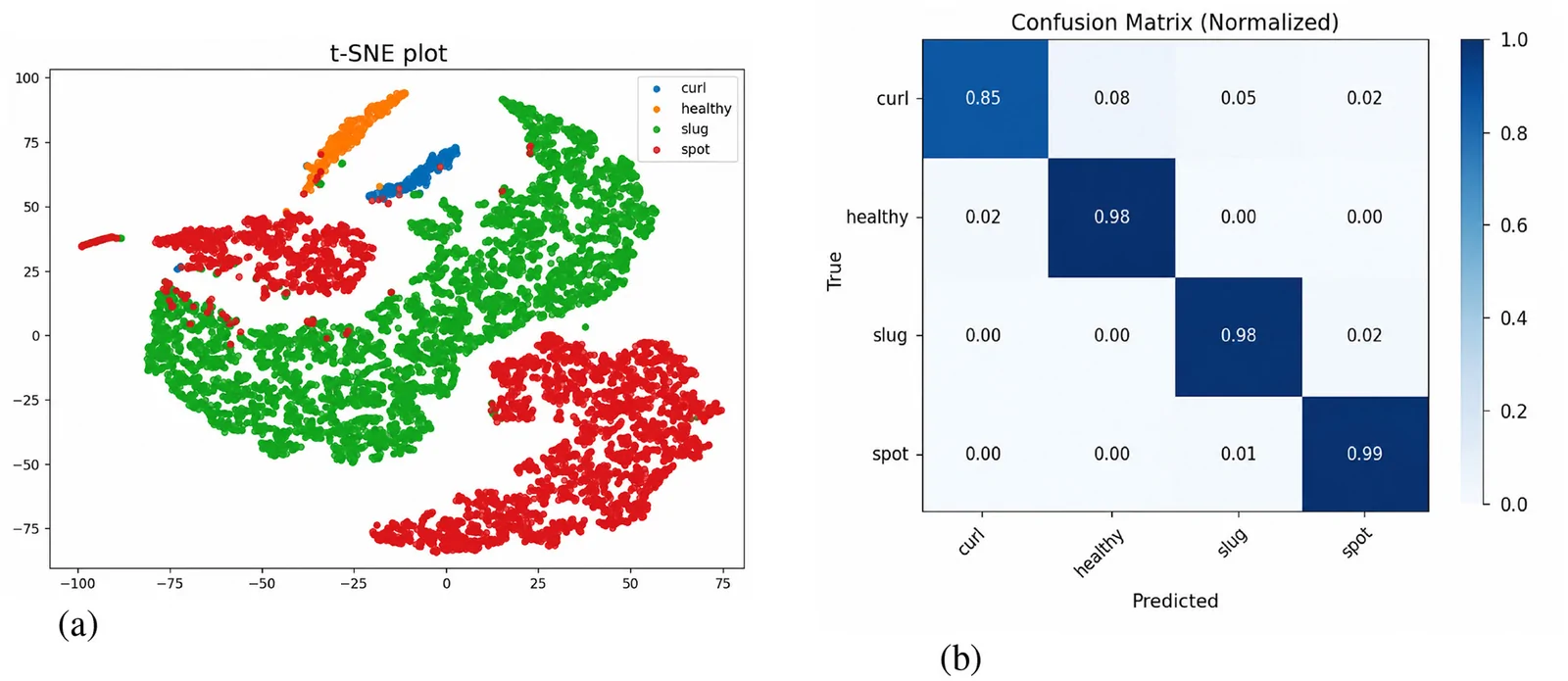
Embedding-space and prediction-level analysis of the proposed framework. **(a)** t-SNE visualization of test embeddings. **(b)** Normalized confusion matrix.

As shown in Figure 5a, the learned embeddings form compact and clearly separated clusters for the four classes. This indicates that the proposed similarity-aware objective encourages intra-class compactness while reducing overlap between visually confusable categories. The separation between clusters provides embedding-level evidence that the model learns discriminative representations rather than relying only on final classification scores.

The normalized confusion matrix in Figure 5b provides prediction-level support for the same observation. The strong diagonal dominance shows that most samples are correctly assigned to their corresponding classes, with only limited confusion between categories. This behavior is consistent with the high macro F1-score reported in Table 2 and the improved class-wise F1-scores shown in Table 4. Together, the t-SNE visualization and confusion matrix demonstrate that the proposed framework improves both the structure of the learned embedding space and the reliability of final class predictions.

### 6.6 Limitations

The experimental results show that addressing inter-class similarity only at the feature or loss level may be insufficient when the input image contains mixed visual information. The ablation study demonstrates that CNN and transformer features are individually useful, but their direct combination does not provide a major improvement. Similarly, applying the similarity-weighted contrastive loss without instance-level localization does not improve performance. This suggests that, for real-field plant disease images, the quality of the input representation is a critical factor. By first localizing disease or healthy leaf regions, the proposed framework reduces background influence and co-occurring visual patterns before feature learning. The similarity-aware contrastive objective can then be applied to the resulting embeddings to improve class separation.

However, the results should be interpreted within the scope of the current study. First, it was only tested on the DiaMOS Plant pear leaf dataset. However, its applicability to other crops, disease types and larger multi-class data sets is yet to be proven. Evaluation on independent datasets with different imaging conditions would be a better assessment of generalizability in the future. Secondly, there is significant imbalance in the dataset, especially between the healthy and curl classes. While macro F1-score and per-class results offer a more balanced evaluation, the number of minority class samples may impact the stability of the performance estimates. Future assessments might become more reliable with more varied samples and more sophisticated data-generation plans for underrepresented classes.Thirdly, the quality of the classification stage is dependent on the quality of the previous localization stage. The inadequate or misleading visual information due to incorrect or incomplete localization can influence the end result of the prediction made by the classifier. Lastly, the disease regions which were used to create the instance-level dataset were manually re-annotated from the original images. These annotations are of varying quality and consistency, which can affect the localized samples and thus the learned representations. Further research may explore the framework with more extensive independently annotated data sets and varying field conditions.

## 7 Conclusion

This study presented an instance-aware and similarity-aware framework for plant disease classification under real-field imaging conditions. The proposed method addresses inter-class visual similarity from two complementary perspectives. First, disease or healthy leaf regions are localized before classification, allowing the model to learn from instance-level patches rather than complete images containing mixed background, healthy tissue, and multiple symptomatic regions. Second, dynamically updated class prototypes are used to identify visually confusable disease classes, and a Similarity-Weighted Contrastive Loss is applied to strengthen separation between difficult class pairs in the embedding space.

Experimental results on the re-annotated DiaMOS Plant leaf dataset show that the proposed framework achieves 97.49 ± 0.73% accuracy and 94.82 ± 0.81% macro F1-score across the five fold models on a locked hold-out test set, and 98.0% accuracy and 93.9% macro F1-score for the logit-averaged ensemble, outperforming the reimplemented inter-class similarity methods evaluated under the same experimental protocol. The ablation study indicates that the largest performance gain comes from reformulating the task as instance-level disease-region classification, while the t-SNE visualization and confusion matrix further support the improved separability of the learned feature space. Future work will focus on validating the framework across additional crops and larger multi-class datasets, improving the robustness of the localization stage, and exploring adaptive strategies for selecting visually confusable class pairs during contrastive learning.

## Data Availability

The original contributions presented in the study are included in the article/supplementary material, further inquiries can be directed to the corresponding authors.
